# Toward Chromoselective Transformations in Biological Systems: Perspectives and Challenges

**DOI:** 10.1002/anie.202526148

**Published:** 2026-06-10

**Authors:** Nadja A. Simeth

**Affiliations:** ^1^ Department of Chemistry Institute For Organic and Biomolecular Chemistry University of Göttingen Göttingen Germany; ^2^ Cluster of Excellence “Multiscale Bioimaging: from Molecular Machines to Networks of Excitable Cells” (MBExC) University of Göttingen Göttingen Germany; ^3^ Department of Chemistry – Ångström Laboratory Uppsala University Uppsala Sweden

**Keywords:** chemical biology, photocages, photochemistry, photoswitches

## Abstract

Addressing small‐molecule chromophores with bioactive properties has been established as an effective means of controlling biological systems for several decades. However, the complexity of in vitro and especially in vivo systems cannot be matched by a single wavelength of light (de)activating one kind of chromophore. Consequently, developing methods to combine and individually control multiple chromophores within the same system is of high importance. An increasing number of strategies have become available relying on the chromoselective transformation of small molecules. This article features the chromoselective control of photoswitches, photoclick chemistry, and photocages, and highlights perspectives and future challenges to be overcome in the context of biological systems.

## Graphical abstract

Controlling biological systems with small‐molecule chromophores has evolved into a powerful strategy and is applied from chemical biology to medicine. However, the complexity of in vivo systems cannot be matched by a single wavelength of light. Developing methods to combine and individually control multiple chromophores is crucial. Here, the chromoselective control of photoswitches, photoclick chemistry, and photocages, and their perspectives and future challenges for biological systems are discussed.

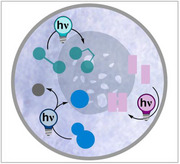

## 1 Introduction

External stimuli are means to gain control over biological systems and processes by precisely activating or deactivating small molecules, biomacromolecules, amoung others [1, 2, 3, 4]. Employing light as an external stimulus has several advantages: photons are unparalleled fast, they can be easily applied or removed with high spatial resolution, they are biorthogonal and do not cause harm to cells, tissue, or living organisms in most scenarios, and their number can be dosed by adjusting light intensity and irradiation time [1, 2, 5, 6, 7]. Naturally, a plethora of light‐responsive small molecules and methods have been developed to exploit the advantageous properties of photons to probe biological systems or develop light‐responsive drug‐delivery systems and pharmaceuticals [2, 8, 9, 10, 11].

**FIGURE 1:**
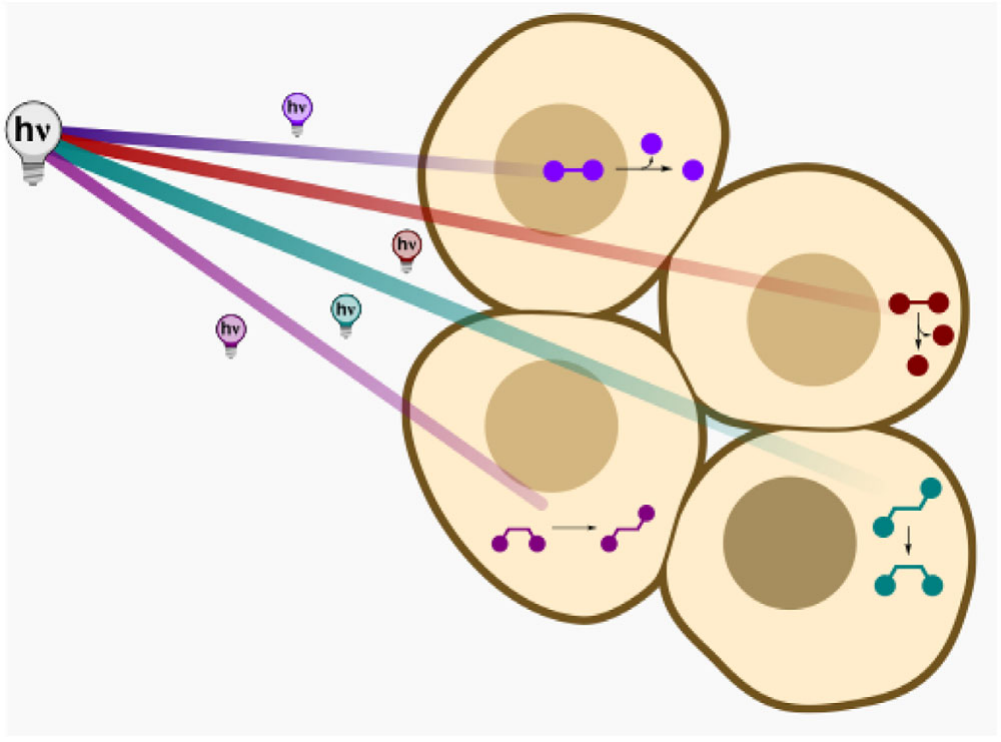
In chromoselective photochemistry, individual transformations of small molecules, such as photoswitches, photoclick agents, and photocages, can be addressed individually in the presence of each other, combining spatiotemporal resolution of light and, beyond that, its discrete energies to trigger selective processes.

Probably the most appealing characteristic of light as a stimulus is that every color, that is, wavelength, is equivalent to a defined energy and thus has the potential to selectively trigger a matching electronic transition in a molecule in the presence of other chromophores. This property allows, in theory, to initiate a whole set of different photochemical processes individually in the same system at selected time points and areas and builds the foundation of precision photochemistry (Figure 1) [12]. Increasing the number and complexity of stimuli‐controlled probes will pave the way to novel ways of studying biological processes, coming closer to the complex, multi‐layer regulation of natural processes in cells and whole organisms.

While this so‐called *λ*‐orthogonal or chromoselective (de)activation of diverse sets of compounds is appealing, the development of more than pairs of molecules or transformations is practically hampered by several molecular and technical challenges that need to be overcome. These limitations include the tuning of absorption spectra, the choice of suitable light sources with defined and, ideally, narrow emission bands, the screening and benchmarking of (wavelength‐dependent) quantum yields and photoconversion cross‐sections, among others.

In this review, examples of different chromoselectively addressable molecules and transformations, with a focus on biological systems, will be highlighted and discussed alongside their current limitations and future challenges to be overcome to unlock the full potential of chromoselective photochemical probes for biological processes.

### 1.1 Chromoselective and *λ*‐Orthogonal—From Discovery and Terminology to Precision Photochemistry

The term “λ‐orthogonal” was introduced by Bochet and coworkers in the early 2000s, when describing the independent release of two photolabile protecting groups (PPGs, see Section 4) [13, 14, 15, 16, 17]. Originally, it was used to describe a set of PPGs that could be addressed individually in the presence of each other [7, 15, 16]. Over the years, the term became more broadly used also for other light‐sensitive molecules, such as photoswitches [18] and photoclick reagents [19, 20]. The high application potential of *λ*‐orthogonally controllable chromophores for a broad range of scenarios has driven the need for designability of pairs or sets of molecules. In 2022, Heckel and coworkers made the first efforts to make the combination of various light‐sensitive molecules more predictable. Specifically, they defined the degree of chromatic selectivity *η*(*λ*) as a fraction between the desired light‐generated product in relation to all light‐generated products at a given wavelength λ [21].

The wavelength dependency of *η*(*λ*) is caused by several factors. First, following the first law of photochemistry [22], molecules that do not absorb at a certain wavelength cannot be excited, and compounds that have a lower molar absorptivity *ε*(*λ*) are less likely to be excited than those with a higher one. As a direct consequence, the complementarity of the UV–vis absorption spectra of a set of chromophores can be used to estimate whether they could be combined within one system and addressed selectively [18]. This approach is typically promising to identify light‐sensitive molecules that can be triggered sequentially, starting from less to more energy‐demanding processes, resulting in “sequence‐specific” or “sequence‐dependent” selectivity [23, 24]. Obtaining sequence‐independent stimulation of light‐sensitive molecules is thus naturally challenging, since organic chromophores tend to have overlapping absorption bands in the higher energy region of the electromagnetic spectrum [21, 25, 26, 27].

The second factor contributing to the wavelength dependency of *η*(*λ*) is that the effectiveness of a photochemical transformation to take place also depends on the (wavelength‐dependent) quantum yield (QY, *Φ*(*λ*)) [12]. The interplay of *Φ*(*λ*) and *ε*(*λ*) is frequently used in the context of PPGs to define the so‐called “uncaging cross section” as the product between the uncaging QY and the molar absorptivity at the wavelength of irradiation (*Φ*_rel_ *ε*(*λ*_irr_)) [6] and is used as a refined estimate of the *λ*‐orthogonality between several PPGs. Importantly, *Φ*(*λ*) and *ε*(*λ*) do not necessarily correlate, so that one cannot assume that wavelength ranges with strongly allowed transitions, that is, high *ε*(*λ*), are automatically the same ranges where photons will effectively transform a chromophore into its photoproduct. Indeed, several groups, including ours, previously exploited this characteristic to realise two preferentially proceeding photochemical processes in the same system despite a limited separation of the UV–vis absorption spectra of the components [21, 27]. To underline the necessity to define the ideal “action regime” of various light‐sensitive molecules (vide infra), Barner‐Kowollik and colleagues systematically recorded “action plots” of diverse light‐sensitive molecules [28], which allowed them to combine chromophores that intuitively might not have been the first choice. Actions plots, however, only provide information in high resolution if the excitation light is monochromatic [12].

The previous two factors, *Φ*(*λ*) and *ε*(*λ*), are both dependent on the nature of the investigated chromophores and the surrounding environment, such as solvent, temperature, and so on. However, in an applied scenario, not only two (or more) molecules and their respective *Φ*(*λ*) and *ε*(*λ*) need to be considered, but the absorption of the resulting ensemble, which might differ from *Φε*(*λ*) if a non‐equimolar ratio is used. Thus, the concentration *c* of the individual chromophores represents an additional factor to be taken into account to achieve full chromatic selectivity *η*(*λ*) in a complex medium [12, 21, 29]. Importantly, the concentration of any component of the reaction mixture may change over time as it is photochemically converted [21, 29]. Thus, the overall absorption of a system will differ between the starting point of the experiment (*t* = 0) and a later time point, making *c* time‐dependent (*c*(*t*)).

Consequently, time *t* itself is a variable to be considered when designing chromoselective processes. With the time‐dependent conversion of one system component upon irradiation, sets of molecules that were considered highly selective at the initial state (*η*(*λ*,*t* = 0)) may be less selective at a later time point, and ultimately, selectivity might be lost fully. The practical consequence of this realization is that high *η*(*λ*,*t*) does likely not correlate with long irradiation times.

Due to their complex interplay in the context of *λ*‐orthogonality, the variables, *Φ*(*λ*), *ε*(*λ*), *c*(*t*), and *t* have been recently recognised as the four pillars of precision photochemistry [12]. Understanding the subtle changes in *Φ*(*λ*), using well‐defined, ideally monochromatic, excitation light to trigger reactions, and recognizing the time point at which a set of chromophores loses its initial chromoselective behavior will allow planned reactions beyond 1:1 mixtures and to establish *λ*‐orthogonal systems by design.

While there is an ongoing debate regarding the semantics in the different fields, in the present text, “λ‐orthogonal” will be used for light‐sensitive molecules that can be triggered sequence‐independently. Processes, that require irradiation with increasingly higher energy light to proceed individually, will be termed “sequence‐specific,” while “chromoselective” will be used for any (set of) process(es), that can be addressed with different colours of light (without necessarily specifying the degree of *η*). The aim is to provide an overview and compare the state of the art in chromoselective control in various light‐sensitive molecules with a particular focus on the perspective of their application in biological systems and potential challenges using illustrative examples.

## 2 Photoswitches

Organic photochemical switches can undergo reversible transformations upon irradiation with (different colors of) light between two (or more) isomers [2, 5, 30]. Appended or incorporated to pharmacologically active molecules or biomacromolecules, they can be used to control the function and activity of the so‐modified molecules by light [1, 8, 9, 10, 11, 30]. One can classify photoswitches by (bond) type of isomerization and by means of stimuli needed to isomerize them [2]. The latter allows to distinguish between T‐type, that is, thermally reversible, and P‐type, that is, photochemically reversible or bistable, photoswitches. P‐type photoswitches are particularly interesting in the context of chromoselective regulation, as already distinguishing between the forward, that is, transforming the stable into the metastable isomer, and the backward, that is, switching from metastable to stable form, are ideally two distinct chromoselective transformations [2, 5, 30].

Indeed, in photoswitches, each photoisomer needs to be treated as an independent chromophore, and thus, not only the selectivity to other compounds but also between the photoisomers needs to be optimized. This aspect is additionally complicated by the correlation between the transformation of one and the simultaneous formation of the other, as well as the limited lifetime *τ* of the metastable isomer(s).

### 2.1 Chromoselective Isomerization in Photoswitchable Dyads

**FIGURE 2:**
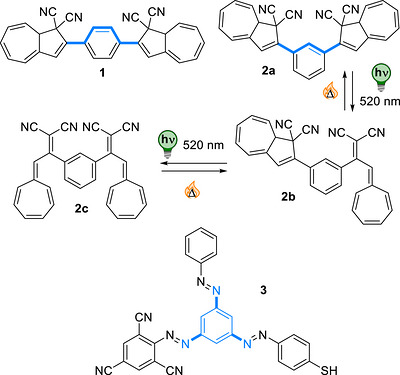
Homo‐dyads based on DHA (top) and azobenzene (bottom) photoswitches.

Nevertheless, efforts have been made to combine several photoswitches within the same systems and build complex, photochromic dyads with chromoselectively switchable components. In this context, the effect of electronic (dis)connection in multimode photoswitches is of key importance. Brøndsted Nielsen and team showed for dihydroazulene (DHA) photoswitch dimers with a *para* (**1**) or a *meta*‐phenylene (**2a**–**c**) bridge (Figure 2) that the *meta*‐connected derivative could be switched between three states through sequential light‐induced ring‐opening reactions into the open vinylheptafulvene (VHF) form [31]. The stepwise isomerization of each chromophore was feasible due to a reduced efficiency of the second ring‐opening reaction (**2b**→**2c**). The authors attribute this behavior to the excitation of **2b**, which has a significant charge‐transfer character, rather than being localized on the DHA as is commonly observed. Also, in the thermal ring‐closure reaction of **2c**, a dependency of both chromophore units was observed. The first ring closure (**2c**→**2b**) showed longer thermal lifetimes than the second one (**2b**→**2a**).

In the case of the *para*‐phenylene connection in **1**, the UV–vis absorption maximum of the doubly ring‐closed DHA–DHA species is bathochromically shifted compared to both an individual DHA and the *meta*‐conjugated dimer **2a**, underlining the electronic connection between both chromophores. Irradiation with light resulted in a ring‐opening reaction; however, the authors noted that they needed to change both the type of lamp and the intensity of irradiation to obtain the VHF–VHF isomer in higher yields, as the ring‐opening quantum yield of DHA **1** seemed to be strongly reduced due to the *para*‐conjugation. As DHA photoswitches were reported to also isomerize efficiently in aqueous medium and buffer and in biologically relevant settings [32, 33], the immediate application of DHA‐based multimodal photoswitches for biological systems can be envisioned.

The type of connectivity (*meta* or *para*) also matters in other homo‐dyads. Wegener, Wachveitl, and colleagues showed that the photophysical and photochemical properties in bisazobenzene (bis‐AB) photoswitches are also strongly influenced [34]. Applying a computational design approach, the same team could show that up to three azobenzene units could be combined, all sharing a common phenyl moiety, when connected in *meta*‐position to each other to electronically decouple the individual chromophores [35]. This principle can even be extended to generate *oligo*‐azobenzene homo‐dyads [36] and was later dubbed as “connectivity *meta*‐rule” [37].

While the photoisomerization of each azo‐switch could thus be addressed independently from the others, their UV–vis absorption bands still overlap, making single‐unit photoswitching not feasible [35]. Thus, the authors aimed to separate the individual bands crucially involved in photoisomerization from each other by introducing suitable substituents [35]. Static quantum chemical calculations allowed the team to identify meta‐tris‐azobenzene **3** as a proof‐of‐concept compound (Figure 2). Specifically, introducing an electron‐donating SH‐substituent in the *para*‐position of one azo unit shifts its ππ* transition bathochromically and separates it from that of the unsubstituted azo‐phenyl group. Similarly, the CN‐groups in 2, 4, and 6‐position shift the same transition even further toward the red, now predicting the separation of all three ππ* transition bands in the condensed azo‐switch **3**. While the work provides guidance in designing such multi‐functional photoswitches, underlying the value of computation chemistry as a cornerstone in dye development, the design needs to be experimentally studied regarding the extent of photoswitching, its efficiency, and the thermal stability of the meta‐stable isomers in different solvents. Especially, the SH‐group might open additional *Z* to *E* isomerization pathways via tautomerization as previously described for various azo‐based photoswitches [38, 39], potentially hampering application in biologically relevant aqueous medium.

**FIGURE 3:**
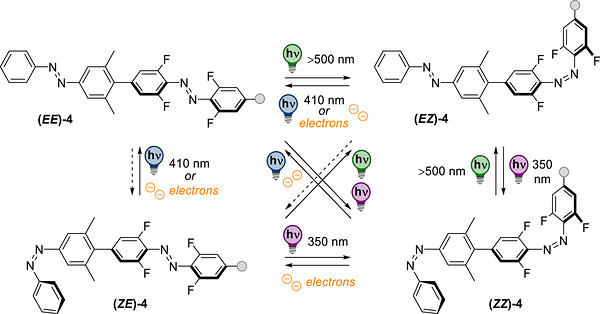
The ortho‐methyl substituents in azobenzene 4 forced the two para‐connected chromophores out of plane and thus disconnected their responsiveness to light. This approach allowed to generate multiple isomers individually through chromoselective control and electrochemical stimuli.

Bléger and coworkers used substituent effects not only to separate transition bands, but to force two connected azobenzenes out of the same plane [40]. This approach allowed the authors to electronically disconnect two chromophores even when connected in *para*. Specifically, they built the dimeric azobenzene **4** consisting of one classical azobenzene and one tetra‐*ortho*‐fluoro‐substituted one (Figure 3). The tetra‐*ortho*‐fluoro substituents led to a sufficient band separation of the nπ* bands of both the *E* and the *Z* isomer of the subunit, both from each other and the nπ* transition of the classical azobenzene moiety. This design allowed for reversible switching of the visible‐light responsive unit with the formation of the photochemically generated isomer **(*EZ*)‐4** in ca. 80% with green light, while UV‐light induced *E* to *Z* isomerization and 410 nm light *Z* to *E* isomerization in both components of the dyad to yield **(*ZZ*)‐4** or **(*EE*)‐4**, respectively. To gain additional selectivity over the processes, electrons were chosen as an additional stimulus to facilitate the isomerization of **(*ZZ*)‐4** to **(*ZE*)‐4**. While this strategy proved effective for the presented system, it also highlighted the current limitation of all‐light‐regulated multi‐chromatic systems and potentially hampers their applicability in more complex settings, such as biological environments. Also, the photochemical studies employing UV–vis absorption spectroscopy were performed in MeCN, and the behavior of the molecules, such as the extent of photoswitching, might be affected by changing the solvent to an aqueous medium.

Despite homo dyads being relatively well studied and the positioning of substituents aiding chromoselective control, the selective addressability of the individual parts is often limited. Hybrid dimers and more extended hetero dyads offer additional possibilities for tunability, allowing for chromoselective control.

**FIGURE 4:**
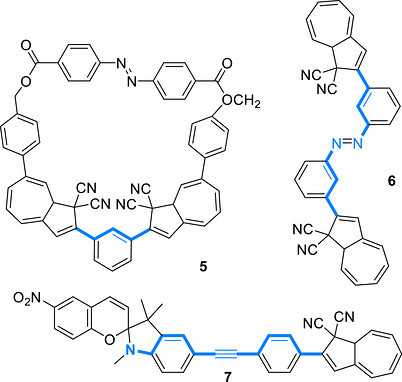
Photochromic dyads connected in *meta*‐position showed increased individual light‐addressability compared to *para*‐connected ones.

Mikkelsen and coworkers, for instance, studied macrocyclic DHA/azobenzene photoswitches by time‐resolved (TD) density functional theory (DFT) calculations [41]. The non‐optimized system, featuring classical azobenzene connected to two DHA units in a macrocycle (**5**, Figure 4), showed strongly overlapping absorption bands of both *E* and *Z* isomers of the azobenzenes with each other and with the main absorption band of the closed form of the two DHA switches, making it impossible to address the light‐sensitive molecules individually. By probing the influence of different substitutes R≠H in the *ortho*‐positions of the azo‐unit, they found that introducing mixed F and Cl substituents led to an improved separation of the ππ* and nπ* bands of the *E* and *Z* photoisomers of azobenzene and the DHA.

By linearly connecting the same components, the authors could gain further insight into the isomerization propensity of the multi‐photochromic assemblies [42]. Together with Di Donato, Cacciarini, Brøndsted Nielsen, and their teams, they synthesized a series of model compounds and investigated the influence of *para* versus *meta* connectivity on the azobenzene core. While azobenzenes with two DHAs in *para*‐position hardly underwent light‐responsive changes upon irradiation with 415 nm light, *meta*‐connectivity on the azobenzene (e.g., **6**, Figure 4) allowed for photochemical isomerization in a stepwise manner; however, at the same wavelength of light. This study highlights the design challenge of achieving chromoselectivity in photochromic dyads.

Spiropyran (SP) photoswitches are known to respond to various stimuli, including light, temperature, and pH [43]. Cacciarini and Brøndsted‐Nielsen hence combined a spiropyran photoswitch with a DHA/VHF photochromic couple in a dyad (**7**, Figure 4) to obtain a system capable of generating eight individual states through different stimuli [44]. While the work on the one hand shows the limitation implied by limited spectroscopic resolution, it also showcases that by increasing the number of stimuli, the complexity of the system can be significantly increased. Generally, this is an important design consideration of orthogonally controlled chromophores; however, in biological systems, drastic changes in pH or temperature from the outside are typically too invasive and cannot be applied.

**FIGURE 5:**
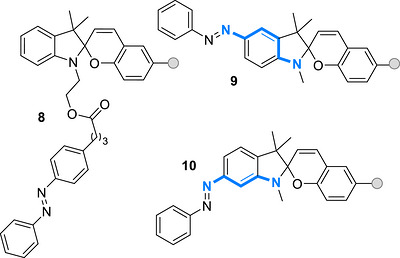
Hybrid dimers connected through linkers or in *meta*‐position showed reasonable chromoselective properties in the isomerization of the individual chromophore units, compared to *para*‐connected ones, even in fused systems.

SP photoswitches have also been combined with azobenzenes to create hetero dyads. Using a short linker, the two units were connected into **8** by Ueda and coworkers (Figure 5); however, both components were addressed with the same wavelengths [45]. More recently, the team of Dreuw, Wachtveitl, and Wegner used the same photoswitches to generate a fused analogue in which one benzene ring of the azobenzene and the benzo‐unit of the indoline part in spiropyran are shared (**9** and **10** in Figure 5) [37]. The authors exploited the *meta*‐rule to decouple the photochemistry of the azo‐unit from that of the spiropyran (compound **10**). At the same time, in **9** the chromophores were conjugated, shifting the lowest electronic transition of the dyad by 60 nm bathochromically compared to **10**. The electronic disconnection in **10** allowed the use of different wavelengths to photoisomerize the two chromophores chromoselectively or simultaneously.

Besides the exemplary systems discussed above, several other photoswitchable homo‐ and hetero‐dyads have been previously studied. For instance, azobenzene and donor–acceptor Stenhouse adduct (DASA) [18], chromene and stilbene [46], azobenzene and oxindole [47], and diarylethenes [48], among others, were combined into hybrid dimers. The constructs typically showed higher levels of *η* when the individual chromophores were connected following the *meta*‐rule or rotated out of plane over an axis and exhibited sufficient band separation. Since the previously discussed systems focus on deciphering the delicate interplay of structural connectivity, substituent effects, and photoisomerization, strategies to further modify the compounds to match solubility requirements for aqueous buffers or cell media are yet to be introduced, following known strategies for photoswitches [2].

### 2.2 Chromoselective Photoswitching in Solution and Biomolecules

When chromophores are not assembled into a dyad, they typically do not influence each other's photoswitching at low concentrations. However, they might still be competing for the same photons, which can be avoided mainly in cases where the band separation is high.

**FIGURE 6:**
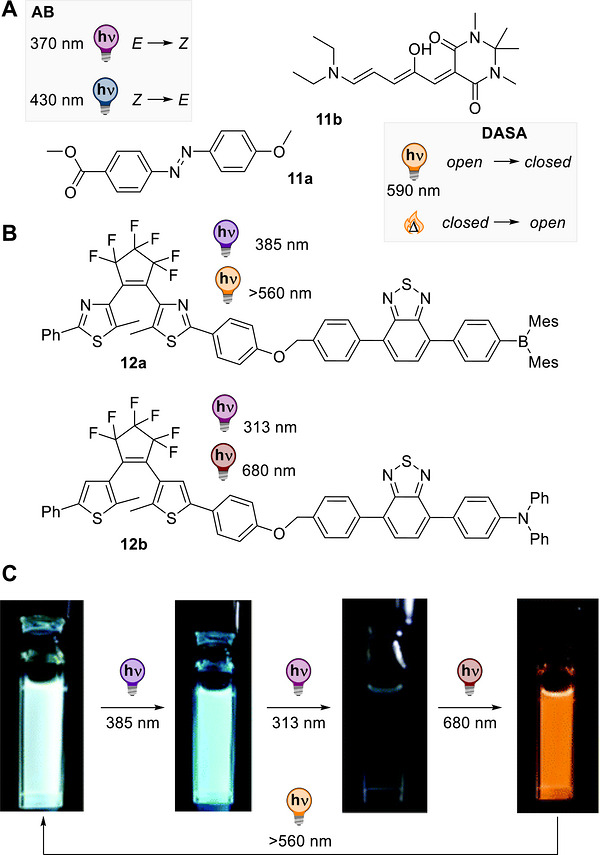
(A) Chemical structures and matching wavelengths of irradiation for the DASA‐AB couples 11a and 11b. (B) Chemical structures of fluorescent DAEs 12a and 12b. (C) Photographs of cuvettes containing a mixture of suspensions of nanoparticles containing 12a (1.3 × 10−5 M) and 12b (1.1 × 10−5 M) NPs after different irradiation intervals [51] (Reprinted with permission from Ref. [51]).

Taking this aspect into account, Szymanski, Feringa, and coworkers combined an azobenzene (**11a** in Figure 6) and a donor–acceptor Stenhouse adduct (DASA, **11b**) in the same system, exploiting the large gap in the UV–vis absorption spectrum of DASAs between 300 and 500 nm [18]. This optical window perfectly accommodates the main electronic transitions of azobenzene photoswitches. The azobenzene could be reversibly switched between *E* and *Z* isomers with 365 and 430 nm light, respectively, while the DASA was ring‐closed by illumination with orange light (590 nm), and the ring‐opening was facilitated thermally. The combination of these stimuli allowed the authors to switch between all four states. Moreover, a dyad consisting of the same components could be moved between an organic (toluene or DCM) and an aqueous phase, depending on the photoisomers obtained after irradiation, using α‐CD to facilitate the phase transfer. The work shows both the potential of *λ*‐orthogonal control in a complex system and exploits the light‐induced change in solubility of the DASA photoswitch. The latter could potentially also be used to transport a photoswitch between lipophilic and hydrophilic compartments in biological systems, similarly as previously realized for an SP photoswitch [49]. Moreover, a DASA–azobenzene couple was recently studied for nonlinear optical applications [50].

Different DASA photoswitches could also be mixed with each other by involving two different acceptor moieties [51]. Since one DASA was responsive to green light irradiation and the other one could be photochemically isomerized through irradiation with red light, two polymersome‐based nanoreactors could be addressed individually. However, the reverse reactions could not be resolved, so that both systems were auto‐inactivated in the dark. Despite the lack of resolution in the ring‐opening reaction of the two DASA molecules, the inherent thermal lability of (dual‐wavelength) photoswitches is a useful design aspect for the remote control of biological systems. The transient activation of an assembly or small molecule within an irradiated area and the subsequent auto‐inactivation upon reaching darkness, facilitated by diffusion, could lead to superb spatial control over activity.

Also, diarylethene (DAE) photoswitches can be addressed chromoselectively to some extent through synthetic modifications. Ishida et al. could generate the four states of two different molecules (**12a**,**b**, Figure 6) and apply these to tune the fluorescence of nanoparticles [52]. While the fluorescence photoswitching showed high contrast and the observed emission could be changed between four different colors, the system appeared to be limited to sequential photoswitching, always addressing first the bands of lower energy in both the open and the closed photoisomers. Moreover, the solubility of the compounds in aqueous media will likely be limited without the introduction of further solubilizing groups [2].

**FIGURE 7:**
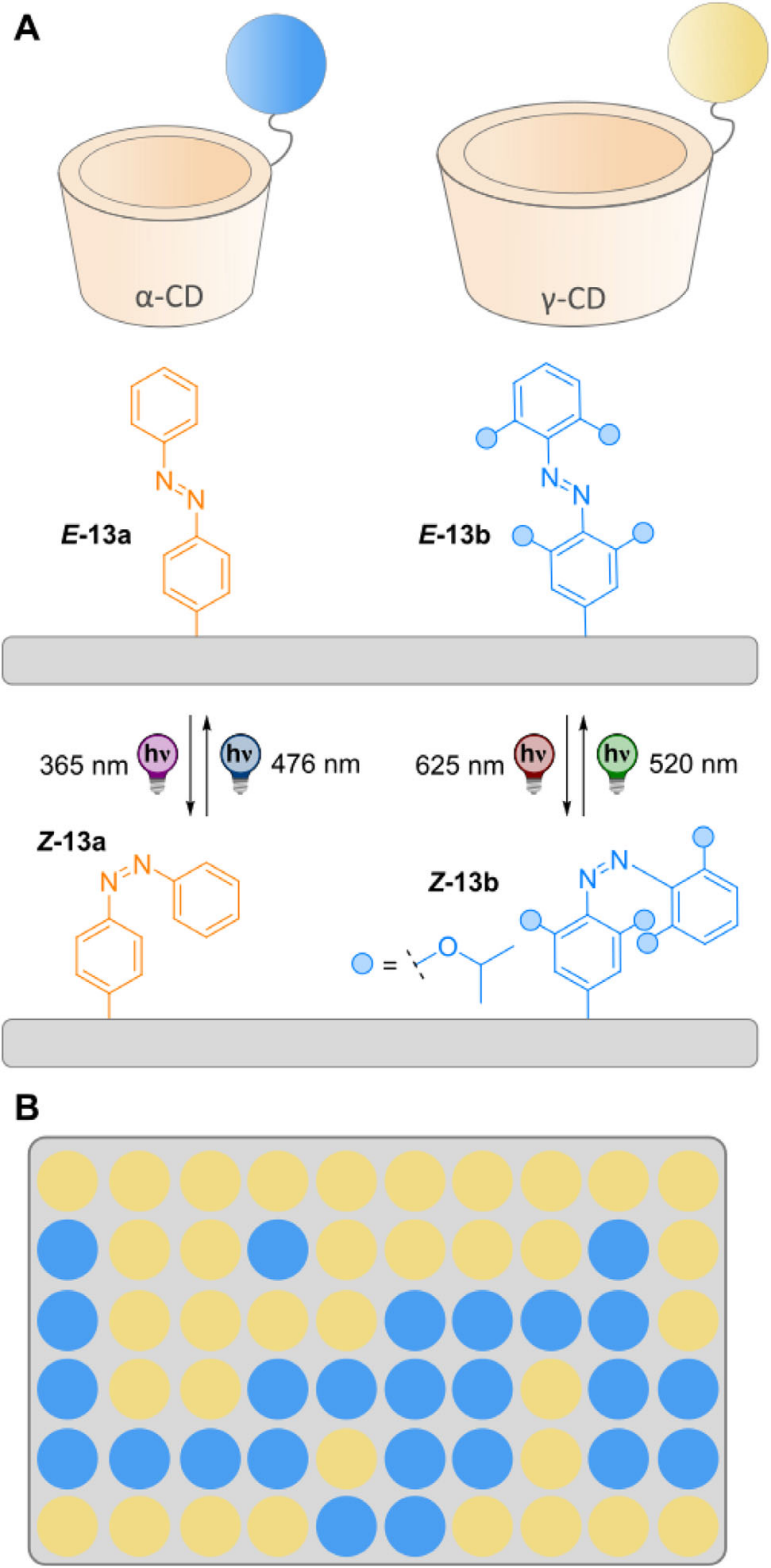
(A) Representation of surface‐immobilized azobenzenes 13a and 13b and their photoisomerization. The different sizes of 13a and 13b allow for binding to fluorescently labelled α‐CD or γ‐CD, respectively, of only the *E*‐isomers. (B) Representation of a patterned surface due to individual isomerization of the two ABs and binding to differently fluorescently labelled CDs.

Wu and coworkers combined a classical azobenzene photoswitch **13a** with a tetra‐*ortho*‐*^i^* PrO‐substituted derivative, **13b** (Figure 7A) [53]. On the one hand, the chosen substitution pattern allowed for selectively addressing each of the four photoisomers with individual colors of light, generating a four‐state photoswitchable system operated with 365, 470, 530, and 625 nm light. Starting from both *E* forms, irradiation with UV light generated 100% ***Z*‐13a** and 75% ***E*‐13b,** with ***Z*‐13b** being formed to 25% in an unintended background reaction through excitation of the ππ* transition of both photoswitches. Further irradiation with red light enriched ***Z*‐13b** up to 60% by now addressing the nπ* transition of the chromophore, while **13a** stayed unaffected by this wavelength. Alternatively, irradiation with green light induced the *Z*→*E* isomerization of **13a**, enriching ***E*‐13b** to 80%, and simultaneously the *E*→*Z* of **13b**, yielding 80% *Z* isomer. Blue light irradiation can be used primarily to induce the *E* form of both photoswitches. In this manner, the authors could indeed demonstrate that four different colors of light can generate four distinct photostationary state distributions, each favoring a different combination of photoisomers.

Next, the two differently large photoswitches **13a** and **13b** were combined with differently sized cyclodextrins (CDs) to obtain two independent host–guest systems, namely **13a**/α‐CD and **13b**/γ‐CD (Figure 7A). While the azobenzene guests could be accommodated by their individual host in the *E*‐forms, the *Z*‐isomers were too large and dissociated. Through labeling each CD with a differently colored fluorophore, the chromoselective control over the surface‐immobilized photoswitches could be applied to generate various colored patterns with four colors (Figure 7B).

The *E*/*Z* isomerization of ABs can also be used to control DNA self‐assembly. For instance, Famulok and coworkers introduced an arylazopyrazole (AAP) photoswitch nucleic acid building block that could be readily introduced into synthetic DNA and RNA [54]. In the *E* form, such nucleic acids can undergo π‐stacking interactions with a complementary strand to form a self‐assembled 3D structure. Upon irradiation with light and accompanied *E*→*Z* isomerization, the π‐interactions are interrupted, and the strands dissociate.

**FIGURE 8:**
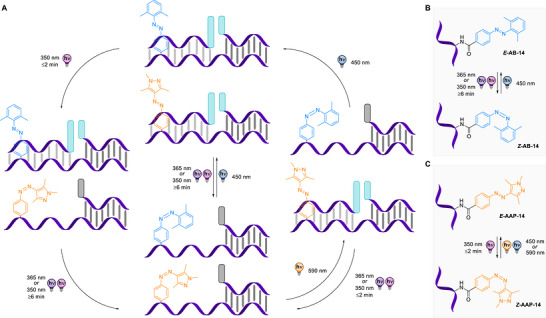
(A) The combination of AB and AAP‐containing nucleobases allowed their incorporation into synthetic nucleic acids. Only the *E*‐isomers led to strand association, facilitating the light‐control of the self‐assembly between two strands and a catalytically active G‐quadruplex domain (gray bar inactive, green bar catalytically active. (B) Representation of the photoisomerization of the light‐responsive nucleobases.

Together with Valero and their team, Famulok and coworkers later used a combination of nucleic acids containing AAP or classical AB, respectively, to chromoselectively control the assembly of a G‐quadruplex that had DNAzyme activity (Figure 8) [55]. The authors positioned the azobenzene‐based nucleic acids in such a way that, upon photochemical isomerization to their respective metastable forms, the strands not only dissociated but also lost the DNAzyme's catalytic center. As the AAP photoswitch isomerizes more efficiently upon irradiation with UV‐A light than the AB‐based one, the *E*→*Z* isomerization of the two compounds could be temporally resolved. Irradiation with 590 nm exclusively isomerized the AAP from *Z* to *E*, while blue light irradiation isomerized both switches from *Z* to *E*. This combination of colors gave a total of four different states of the system, which controlled the catalytic activity of the DNAzyme. The same system was further used to develop a light‐controlled DNA‐based molecular logic system showcasing the potential of the presented concept as a basis to build complex molecular circuits.

Following a similar concept, but combining AB and *para*‐MeS‐AB in two different DNA strands, the authors devised a non‐autonomous DNA walker [56]. With *para*‐MeS‐AB having a UV–vis absorption maximum bathochromically shifted compared to AB, and higher photoisomerization efficiencies, the DNA walker “leg” containing these nucleic acids could quickly undergo *E*→*Z* isomerization with 385 nm light irradiation, and thus, the strands dissociated. Then, treatment with 365 nm addressed both photoswitches and led to the sequential formation of *Z*‐AB, dissociation of the second “leg.” Irradiation with 385 nm light generated again the *E*‐AB but kept the *Z*‐MeS‐AB, and subsequent irradiation with 450 nm regenerated the all‐*E*‐state. The authors coupled this molecular motion and the associated strand association/dissociation process to DNA‐strand replacement in such a way that the formation of the new dsDNA was coupled to a movement of the DNA‐hybrid construct on a surface, allowing the system to “walk.”

Despite the high level of control shown in these works, the limited band separation of the azo‐switches did not allow for a fully *λ*‐orthogonal generation of all states by light. For the DNAzyme, the key aspects that allowed the generation of four different states were, on the one hand, the band separation of the nπ* bands of the *Z* isomers to selectively address *Z*‐AAP with green light, and on the other hand, the higher photoisomerization quantum yield (QY) of *E*‐AAP compared to *E*‐AB, which made it possible to favor *E*→*Z* isomerization of AAP over AB kinetically. In the case of the DNA walker, the authors used the combination of the band separation of both *E* isomers and the higher photoisomerization quantum yield of the MeS‐AB to isomerize it over AB selectively. Additionally, the overlap of the nπ* of *Z*‐AB with the ππ* of *E*‐MeS‐AB facilitated the selective isomerization of AB from *Z* to *E*.

Addressing all individual isomers fully *λ*‐orthogonal was due to the large overlap of the bands not yet achievable, but would unlock more complex DNA‐hybrid designs in the future. Moreover, the same approach could be extended to other biomacromolecules, including light‐responsive amino acids, sugars, and fatty acids, to push the current limits of chromoselective processes in biology.

### 2.3 3D Resolution With Two Wavelengths

While many of the photoswitchable systems discussed thus far have focused on developing *λ*‐orthogonal processes that can be controlled independently of each other, dual‐wavelength photochemistry can also be leveraged to couple two photochemical processes. In this manner, the desired photochemical transformation proceeds only in areas where both reactions are activated, that is, where the irradiation wavelengths overlap, leading to a resolution in 3D space comparable to 2P activation of PPGs (vide infra).

**FIGURE 9:**
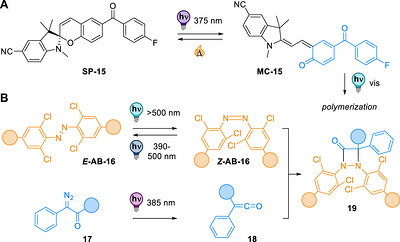
(A) spiropyran‐photoinitiator hybrid ring‐opens and becomes active under UV irradiation, while the initiator is triggered exclusively from **MC‐15** upon visible light illumination. (B) Only when photochemically generated molecules ***Z*‐AB‐16** and ketene **18** meet in the same 3D volume, ligation product **19** is formed.

Recently, Hecht and coworkers combined a spiropyran (SP) photoswitch and a photo‐polymerization initiator in one molecule (Figure 9A) [57]. In the ring‐closed SP‐form of the switch **SP‐15**, the benzophenone moiety is masked, locking the polymer‐initiator. Upon irradiation with 375 nm, the ring‐open merocyanine (MC) **MC‐15** is formed, revealing the benzophenone, which can locally initiate the formation of a polymer from a pentaerythritol tetraacrylate‐containing resin in the presence of visible light. Importantly, the MC form is thermally labile (*τ*_1/2_  =  6s), immediately giving back the SP isomer when the 375 nm light source is switched off. Thus, photopolymerization can only occur when both 375 nm (to switch to MC and unmask the benzophenone) and visible light (to excite the benzophenone) irradiation is applied simultaneously at the same location.

The team of Truong and Barner‐Kowollik, on the other hand, used an azobenzene photoswitch directly to undergo a photoligation reaction, only in one isomer (Figure 9B) [58]. Irradiation with <500 nm isomerized ***E*‐AB‐16** to ***Z*‐AB‐16**, while illumination with 385 nm photochemically generated ketene **18** from diazo **17**. Only when both transient molecules were generated in the same 3D volume could a covalent bond be formed between them, yielding **19**. By conducting a thorough action plot study, the authors identified suitable irradiation wavelengths that chromoselectively targeted only one of the two light‐sensitive processes. This aspect is crucial to avoid background reactions outside of the dually irradiated volume.

As this type of photochemical reactivity enables high precision in 3D space, it is highly attractive for biological studies, as it would lead to unmatched spatial resolution in cells, tissues, or organisms. Comparable efforts of PPGs are discussed below.

### 2.4 Photoswitches in Biological Context—A Challenge for Chromoselective Systems?

While several different photochromic couples could be combined in an in vitro setting, not all the above‐discussed systems are likely to be readily applicable in cellulo or in vivo. Photoresponsive systems based on organic chromophores face the same challenges as small‐molecule drugs and organic tools for chemical biology [59]. Thus, additional adjustments might be required to ensure compatibility with biological systems. On the one hand, the influence of aqueous media and the high salt content of buffers and cellular media on the solubility of the photoswitches, their photochemical behavior, and the thermal stability of the metastable isomers needs to be considered. It has been shown that motifs from bioactive molecules in combination with photoswitchable entities can indeed be directly used for bioactivity studies and even in vivo [9, 60]. For instance, the abovementioned (hetero)azobenzenes **AB‐14** and **AAP‐14** still performed reliably when incorporated into DNA, influencing the double‐strand hybridization of the biohybrid constructs through light‐mediated structural changes [54, 55, 56]. The pairs of chromophores could be toggled between the respective photoisomers without dramatic fatigue several times [54, 55]. Thus, an application in a living biological environment seems within reach. However, photochromic diads, such as molecules **1**–**10** (vide supra), will likely require the introduction of additional solubilizing groups to be water‐compatible in the first place.

While water‐solubility has been investigated for many photoswitches and was extensively reviewed elsewhere recently [2], the complexity of biological environments poses additional challenges, beyond the solvent. For instance, the presence of extra‐ and intracellular proteins, cellular antioxidants, like glutathione, in high concentrations, and the different conditions in different cellular compartments, as well as intracellular delivery, need to be considered.

Moreover, photoswitching induces a change in structure and polarity that can impact the solubility and permeability of a switch. For instance, the change between spiropyran and the zwitterionic merocyanine was shown to influence membrane partitioning in a liposome [49]. Also, the hydrophobicity of the organic core structure of many photoswitches can allow them to bind to proteins in a non‐selective, supramolecular manner. DTEs, for example, could form supramolecular complexes with serum albumins, impacting their photochemical properties and even imprinting the chiral environment posed by the protein on the ring‐closed photoproduct [61, 62].

**FIGURE 10:**
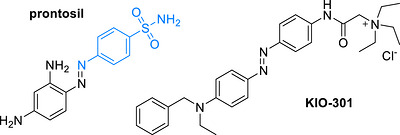
Chemical structures of pronotosil, one of the first antibiotics, and **KOI‐301**, a molecule under clinical investigation to cure blindness. While pronotosil acts as a prodrug, realizing the sulfonamide displayed in blue after reduction of the azo‐bond, **KOI‐301** is relaying on photoswitching as mode of action.

Especially, azobenzenes are known to be unstable toward glutathione (GSH) and other cellular reductants and may potentially degrade through reduction of the central azo function to the corresponding hydrazine or anilines. In particular, GSH, ascorbic acid, Cys, and also dithiothreitol (DTT), used in protein biochemistry, have been shown to transform azobenzenes into hydrazines in the range of minutes in vitro [63, 64]. However, not all azobenzenes are equally sensitive to reduction in biological medium [63, 64, 65] or are applied in a context where high amounts of reducing agents are present. The difference in performance can be illustrated by comparing prontosil, the first synthetic antibiotic [66], and **KIO‐301**, an azobenzene photoswitch that is currently in clinical phase II for vision restoration (Figure 10) [67]. While the first one acts as a prodrug releasing the bioactive reduced form (moiety in prontosil highlighted in blue in Figure 10), **KIO‐301** is a true photopharmacological agent. The photopharmacophore can block voltage‐gated ion channels upon light irradiation and quickly isomerizes back thermally to become readily available for the next light pulse. **KIO‐301** is part of a large family of photoswitchable ion channel modulators for healing blindness [68, 69, 70, 71, 72, 73, 74]. Many of the compounds perform well even in animal models, and all rely on robust photoisomerization for their biological activity.

Indeed, many photoswitches could already be successfully applied in cellulo and in vivo, showing promising performance. For example, DTE photoswitches can be used as blinking dyes in super‐resolution microscopy when solubilizing groups are attached [75], and are even compatible with live cell imaging [76]. Stiff‐stilbene‐based G‐quadruplex binders showed selective anticancer and antiparasitic activity in cell studies [77], while azobenzene‐based microtube‐stabilizers [78] and photolipids [79, 80] have found broad application from model systems to cells and animal models [81], to only mention a few examples.

In short, many classes of organic photoswitches have been extensively investigated for their applicability in biological environments both from a fundamental point of view and in applied scenarios, including cell and animal models, with azobenzene‐based **KIO‐301** having advanced to clinical studies. Also, successful work has been done to show that precision photochemistry can be performed in buffered media to impact the structure and function of biomolecules. Together, these studies will serve as a foundation for establishing also controllable sets of photoswitches showing high *η* for advanced biological studies.

## 3 Photoclick Reactions and Photocycloadditions

Photoclick or photochemical click reactions are efficient, simple organic transformations that connect two molecular entities with high atom economy, initiated or facilitated through irradiation with light [19, 20]. They can be divided into different classes based on either structural features or their reactivity with many examples involving photocycloaddition reactions. They are mainly mediated through the direct irradiation of the (pre)click handle; however, more recently, indirect excitation through photosensitization has become more frequent, with the aim of shifting the wavelength of excitation further to the red and near‐infrared (NIR) regions of the electromagnetic spectrum [20, 82].

Compared to photocages or photoswitches, photoclick chemistry has only emerged recently in chemical biology and imaging. However, the orthogonality of different click reactions to each other [83, 84] and structural diversification through synthetic chemistry make it possible to assign one type of click reaction to one color of light to achieve chromoselectivity. Frequently used reactions are (reversible) [2+2] and [4+4] photocycloadditions or photo‐enol reactions as they show clean conversions and suitable tunability and orthogonality between individual chromophores [85, 86, 87]. While the applications in biology are yet sparse compared to, for instance, the chromoselective control of polymer properties, the impact these chemistries had on the latter indicates the potential that tailored systems might have in several disciplines.

### 3.1 Photo‐Enol Reactions

**FIGURE 11:**
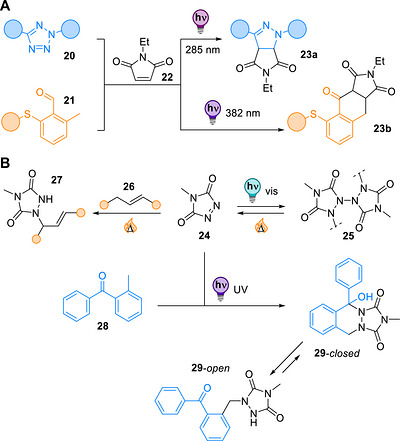
(A) Chromophores **20** and **21** reacted with maleimide **22** at different wavelengths of light into distinct products. (B) Depending on the (absence) of different light stimuli, **24** reacted selectively to either **27**, **25**, or **29**.

For instance, Blinco, Barner‐Kowollik, and colleagues combined a photochemical tetrazole ligation and photoenol ligation in the same system, addressing each light‐sensitive molecule with individual colors of light [88]. Specifically, the tetrazole **20** (Figure 11A) could be irradiated with 285 nm LED light to undergo a photocycloaddition reaction with a maleimide (**22**) to form **23a** under the extrusion of N_2_. At the same time, the *ortho‐*methylbenzaldehyde (**21**) reacts with the same maleimide **22** under irradiation with 382 nm light to yield **18b**. This was achieved by both an optimal match of the absorption profiles (*λ*_max_ (**20**) = 285 nm matching a minimum of **21**, *λ*_max_ (**21**) = 382 nm, where **20** is not absorbing) and quantum yields (Φ of **20** is more than 30 times higher than the one of **21**, thus, more than compensating for the overlap in absorption) favoring the intended process over the competing one at each wavelength leading to chromoselective, programmable covalent bond‐formation. Notably, the chemistry proceeded in a water–cosolvent mixture, indicating the potential compatibility of the underlying photochemistry with biological media.

The same strategy was extended to post‐synthetically modify a polymer with two different functionalizing groups, which responded to *λ*‐orthogonal wavelength [88] and to choose the connectivity in a polymer chromoselectively [89].

Chromoselective transformations can also be used to indirectly control thermal processes by using a photochemical (reversible) reaction to deplete the reaction mixture of one reaction partner. In this manner, Barner‐Kowollik and their team used the visible‐light‐induced polymerization of 1,2,4‐triazoline‐3,5‐diones (TADs), such as **24** (Figure 11B), to distinguish between thermal and photochemical addition‐products [90]. In the dark, the TAD polymer **25** would reverse into its monomeric form **24** and further react with alkenes like **26** into the corresponding vinyl adducts **27** or, in the presence of UV light, with acetophenones (e.g., **28**, Figure 10B) into the respective photo‐enol product **29**. Thus, three different reaction outcomes can be achieved from the same reaction mixture depending on the (combination of) (photochemical) stimuli employed.

### 3.2 [2+2] and [4+4] Photoadditions

Another aspect that becomes apparent when discussing photochemical control in biological systems is the concern of phototoxicity of high‐energy light in the UV‐range of the electromagnetic spectrum. Thus, not only is sufficient band separation an important design criterion, but also the energy required to initiate the photoclick reaction [20, 82].

**FIGURE 12:**
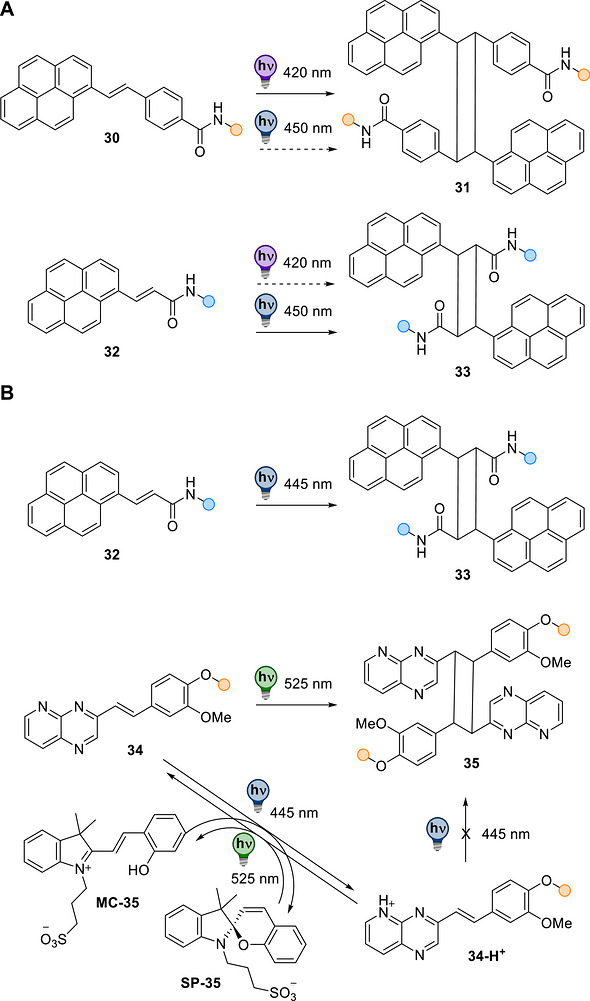
(A) The position of the amid linkage influenced the action regime of **30** and **32** sufficiently to resolve their [2+2] reactions. (B) To independently photoclick **32** and **34** in the same medium, a blue‐light‐responsive photoacid, that is, **MC‐35**, was added to protonate and thus, inactivate **34**, so that only **32** reacted. Under green light, **34‐H^+^** was deprotonated and selectively underwent a photocyclo addition reaction, thanks to the low‐energy absorption band tailing further into the visible light range than **32**.

Especially, [2+2] and [4+4] photocycloaddition reactions typically require irradiation with UV‐light <350 nm. To address this inherent limitation, introduction of electronically non‐innocent substitutes can be employed, such as a triazole group at the 9‐position of anthracene to make a [4+4] cycloaddition reaction sensitive to lower energy light [91] or the attachment of extended π‐systems directly onto the C═C double (e.g., **30** in Figure 12A) involved in the photocycloaddition reaction to readily react to blue light (455 nm) even in the presence of DNA [92].

Kalayci et al. were able to red‐shift the above used styrylpyrene **30** with an amide group on the other side of the C═C bond (i.e., **32** in Figure 12A) to respond to light up to 490 nm [93]. This modification not only allows for a photocycloaddition reaction with unprecedentedly low‐energy light, but also makes it possible to chromatically resolve the photochemical transformation of both chromophores from each other. Specifically, by performing action plots of both alkenes, **30** and **32**, the authors found that 420 nm predominantly addresses the styrylpyrene **30**, while 455 nm leads to a higher reactivity of the acylamidylpyrene **32**. Thus, two different wavelengths of light could be used to address both light‐sensitive molecules in the same system and now use different colors to tune the properties of the hydrogel in which the chromophores were embedded. Moreover, the authors could employ the hydrogel to allow fibroblasts to grow on it and use the light‐induced stiffening of the gel to induce cell detachments, which made it easy to collect and reseed them.

The [2+2] photocycloaddition of **32** induced by 445 nm and the same reaction of a styrylpyrido[2,3‐b]pyrazine (**34**, Figure 12B) could be resolved by introducing a pH‐dependent gating mechanism [94]. To avoid **34** from reacting at the same wavelength as **32** (445 nm), the authors added **MC‐35** as photoacid generator (PAG), which protonated **34** to form the unreactive **34‐H^+^** under the photocycloaddition conditions of the **32**, blocking **34**’s reactivity. Due to the UV–vis absorption spectrum of **34** tailing further towards the red, the [2+2] reaction could also be triggered with green light (525 nm) under which wavelength also **SP‐35** reverted, deprotonating **34‐H^+^** and thus, regenerating the reactive **34**. Thus, two wavelengths could be used to resolve the same reaction type of two different substrates by adding a third photochemical process in a complementary manner.

A key challenge to broaden the application of [2+2] photoclick reactions to biological systems might be the limited water‐solubility of the extended π‐systems, which are used to shift the UV–vis absorption properties to the visible range. Introduction of PEG chains [93] or direct incorporation into biomacromolecules (vide infra) would circumvent this limitation; however, it simultaneously limits the flexibility of the chromophore design. One potential application was shown recently by using a [2+2] photoclick reaction to generate a photoresponsive tag for visible‐light‐induced DNA labeling [95]. A π‐extended styryl unit was used as a handle on a DNA strand and reacted under blue light illumination with a second one, which was labeled with an Atto dye. The reaction proceeded efficiently in aqueous media and even in live HeLa cells, underlining the potential that *λ*‐orthogonal processes, such as those introduced previously, could also be applicable to cellular and eventually complex living systems in the future.

**FIGURE 13:**
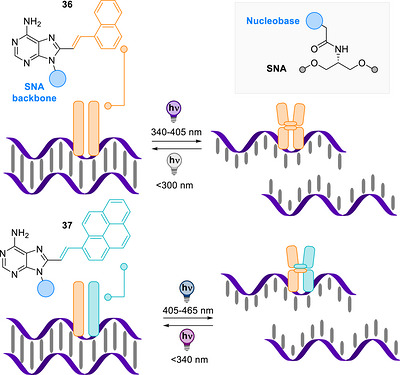
[2+2] photocycloaddition substrates 8‐naphthylvinyl adenine (**36**) and 8‐pyrenylvinyl adenine (**37**) were embedded as pairs into serinol nucleic acid (SNA) to hybridize with RNA as monomers and to induce dissociation after irradiation. Due to their compatibility in light‐responsiveness, homo and the hetero [2+2] dimerization of **36** and **37** could be realized chromoselectively [96].

Taking the same class of chromophores one step further, Yamano, Murayama, and Asanuma developed wavelength‐selective photocycloaddition reactions to crosslink DNA‐strands (Figure 13) [96]. To incorporate the light‐responsive entities into nucleic acids, 8‐naphthylvinyl adenine (**36**) and 8‐pyrenylvinyl adenine (**37**) were selected as unnatural, photoresponsive nucleobases. They were embedded into serinol nucleic acid (SNA), which is a xeno nucleic acid with a non‐ribose scaffold, to generate a photoresponsive SNA strand that could hybridize with RNA. Illumination with 340–405 nm triggered a homo [2+2] photocycloaddition of two nucleobases **36** positioned next to each other in the SNA strand. Since the photocrosslinking occurred close to the backbone of the artificial nucleic acid, the double‐strand stability was reduced, and the light‐sensitive SNA dissociated from the target RNA. Subsequent irradiation with light <300 nm reverted the [2+2] reaction and gave the initial SNA‐strand back, so that the overall system became reversible.

Combining a naphthyl‐containing base **36** with a pyrenyl base **37** allowed to trigger a hetero [2+2] reaction with 405–465 nm irradiation, which could be reverted with light <340 nm. As the homo and the hetero [2+2] dimerization can be resolved with different colors of light, a total of four different states of SNA/RNA duplexes could be generated involving four different wavelengths (465, 405, 340, and 300 nm), selectively associating and dissociating the respective targeted strand by irradiation.

**FIGURE 14:**
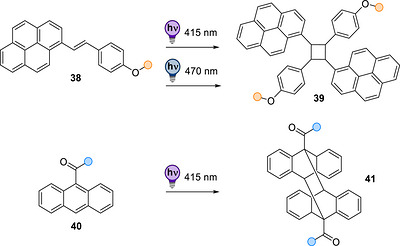
A combination of a [2+2] and a [4+4] photoaddition reactions showing sequential selectivity through irradiation with 470 and 415 nm light [97].

Similar to other click reactions [83, 84], different types of photoclick reactions can also be combined in the same system to enhance the diversity of transformative molecular platforms available. Combining a styrylpyrene‐based one (**38**) with an anthracene side chain (**40**, Figure 14) in the same polymer, the material could be photo‐crosslinked stepwise: The extended π‐system of **38** made it responsive to 470 nm light and triggered a [2+2] reaction, while subsequent irradiation with 415 nm crosslinked the anthracene moieties via a [4+4] reaction [97].

Also, a pyrenylvinyl moiety could be combined with an *ortho*‐methyl benzaldehyde to generate a bifunctional linker, with each end responding to a different wavelength of light, initiating either a [2+2] or a photo‐enol reaction with the same maleimide (cf. **16** in Figure 10) [98]. Moreover, in a bifunctional photo‐enol substrate, the first and the second ligation reaction could be performed sequentially by decreasing the wavelength of irradiation from 385 to 365 nm to accommodate the UV–vis absorption spectrum of the substrate shifting hypsochromically after the first addition reaction [99]. Sequential irradiation could also be used to facilitate photoligation in polymers by first inducing the formation of the polymer backbone by orange light (590 nm) and subsequently linking the fragments by application of blue light (415 nm) using tetrazole‐photoclick chemistry [100].

Photoclick reactions can be utilized for wavelength‐selective chemistry by leveraging a growing toolbox of chromo‐ and chemoselective reagents. While they are rarely used in biological systems so far—potentially due to the limited solubility of the chromophores and their dominant use for ligation chemistry, which cannot be directly coupled to modulating bioactivity—the chemistry is compatible with aqueous systems, and the first examples for controlling biomacromolecules chromoselectively point to their potential. Besides the illustrative examples highlighted above, many classical click reactions can also be light‐controlled by masking one or both click reaction components with photocages. (19, 20) Strategies for using the latter in a chromoselective manner are discussed in the following sections.

### 3.3 Consideration for Photoclick Reactions and Photoligation Chemistry in Biological Systems

Since photoclick reactions can be triggered with photons as biologically begin reagent, their application for bioorthogonal chemistry, for instance, for spatiotemporally controlled protein labeling, appeared natural. Consequently, there was a significant drive in the field to develop novel photoclick reactions and to optimize existing ones to meet the requirements of biological systems [19, 20, 101, 102]. Thereby, the type of photoclick reaction largely determines its immediate applicability. Generally, photoclick chemistry can proceed by reaction of the chromophore from the excited state, by generating a highly reactive intermediate with light, or by photochemical cleavage of a PPG from a “classical” click agent that readily reacts from the ground state [19, 20].

In the latter case, the PPG is used to inactivate an already well‐established click reagent, so that light illumination gates its reactivity. Thus, this type of photoclick reaction can be rapidly adapted to respond to different wavelengths of light by employing PPGs that were optimized to work chromoselectively. However, cleaving off the PPG will produce a photoproduct as a side product, which may potentially impair the biocompatibility of the photoligation. These aspects, together with considerations on phototoxicity and solubility, are discussed in greater detail in Section 4. Here, a few illustrative examples of PPG‐mediated photoclick reactions already working in biological systems are mentioned.

**FIGURE 15:**
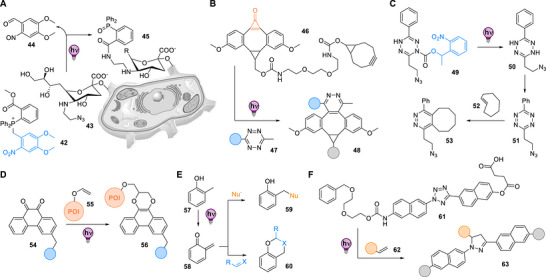
(A–C) Light‐induced cleavage of ONB and CO, respectively, to induce a Staudinger–Bertozzi (A) [103], tetrazine‐alkyne (B) [105], or tetrazine‐alkene (C) photoclick reaction [106, 107]. (D) The excited state formed from 9,10‐phenantroquinones (PQs) under irradiation undergoes [4+2]‐photo‐cycloadducts with alkenes [110, 111]. (E) Photoenol formation and reaction with nucleophiles or alkenes [112, 113]. (F) Tetrazoles form reactive intermediates for photoclick chemistry with alkenes [101].

For instance, by the introduction of an *ortho*‐nitro benzene (ONB) photocage on a tri‐aryl phosphine reagent, a Staudinger–Bertozzi ligation with azidoglycoproteins on the surface of living cells and in zebrafish could be spatiotemporally controlled by light (Figure 15A) [103]. Moreover, cyclopropenone inserted into a benzo‐substituted eight‐membered ring acts as a photocaged strained alkyne. Thus, compounds, like PEG‐ylated **46** displayed in Figure 15B, can readily react with azides [104] or tetrazines incorporated in proteins via genetic code expansion, even in living cells [105]. Also, the reactive counterpart, that is, the tetrazine, could be controlled through cleavage of a PPG from the reduced dihydrotetrazine form, which is readily oxidized by the environment [Figure 15C]. The so‐activated tetrazine could then be clicked to *trans*‐cyclooctene (TCO) on the surface of live cells and later also used to label sections of zebrafish with multiple colors [106, 107]. Alternative means for light‐activation, for example, through photocatalytic activation of tetrazine with red light, were also investigated for in vitro labeling and bioconjugation [108]. However, the applied photocatalyst, methylene blue, is known to generate reactive oxygen species (ROS) [109], and thus an application in living cells or in vivo might need to be evaluated under this aspect.

On the other hand, photoclick reactions involving excited state intermediates, such as photocycloadditions or photo‐enol reactions (vide supra), need to not only show high selectivity toward their click partner, but also react extremely fast before becoming unreactive in the ground state. While achieving selectivity and controlling reaction rates in complex environments like living cells poses a challenge, the short‐lived reactive species generated in this photoclick chemistry offer enhanced spatiotemporal control. Because these species are formed only upon irradiation, their diffusion is limited and ligation occurs exclusively in the illuminated region.

In this context, it was shown that 9,10‐phenantroquinones can fluorogenically form [4+2]‐photo‐cycloadducts with alkenes in vitro and living cells (Figure 15D) [110, 111], despite being known photosensitizers [114]. Also, cresol or hydroxy‐methyl phenol derivatives rearrange into their enol forms under irradiation (cf. **57** to **58** in Figure 15E and Section 3.1). Such structures have been reported to react either with nucleophiles or with C═C or C═X bonds [112, 113]. While the reactivity can be generally tuned by adjusting the chemical structure [115] and protein labeling has been shown [116], in a thiol‐rich environment such as the cell, selectivity toward amino acid side chains or a C═C or C═X click partner will likely still be challenged by the thiol‐quinone methide photoclick reaction with glutathione, potentially impairing the general bio‐applicability of this reaction.

Photoclick reactions generating a reactive intermediate, such as the photoinduced tetrazole‐alkene cycloaddition (Figure 15F), have already found applications for protein labeling in vitro and in vivo [101]. Using π‐extensions, the absorption wavelength of the chromophores could be gradually shifted bathochromically, while the simultaneous introduction of PEG linkers improved aqueous solubility. Importantly, it was shown that ion concentration and pH influence the reaction, with a high Cl^−^ concentration inhibiting labeling of an acrylamide‐encoded sfGFP at pH 7.0 in vitro [117, 118]. Replacing the acrylamide with a strained cyclopropene derivative, the reactivity could be recovered [119] and different variations of the reaction could be realized in living plant and mammalian cells [101, 120, 121]. Incorporating the allyl click handle onto nucleobases, the same type of photoclick chemistry was used to conduct labeling chemistry in living cells and zebrafish embryos in vivo [122]. In this case, it was crucial to introduce a solubilizing sulfonate group onto the photoclick agent.

Taking together the photoclick reactions, which were already successfully applied in living cells and in vivo, and the high levels of chromoselectivity achieved in vitro, wavelength‐selective precision photoclick chemistry in complex biological settings seems within range.

## 4 Photocages

Photolabile protecting groups (PPGs), also called photocages, can be covalently attached, for instance, to small bioactive cargos or functional groups in biomacromolecules, and subsequently removed by light, resulting in on‐demand liberation and often activation of the *caged* molecular unit. A plethora of different PPGs has been developed over the past decades, with an increasing focus on visible‐light responsive representatives and chromoselective processes [6, 123].

Like with the previously discussed chromophores, broad, overlapping absorption bands of most organic chromophores, especially in the UV range, pose a significant challenge in achieving selectively addressable pairs, triplets, and so on, of photocages. Thus, the absorption bands in this spectral range can only be employed to a limited extent to trigger an uncaging process, as addressing the wrong chromophore is probable. Tuning PPGs in such a way that the lowest energetic transitions are shifted into the visible range of the electromagnetic spectrum is a frequently used approach to separate absorption bands and facilitate more chromoselective processes in a sequence‐dependent manner and, when appropriately designed, also in a sequence‐independent way [7].

### 4.1 Sequence‐Dependent Strategies

Engineering chromophores through π‐extension or electronically impactful substituents is a valuable approach to achieve better band separation and move (part of) the UV–vis absorption spectrum into the visible range. Such PPGs can then be combined with unmodified cores to realize selective uncaging sequentially—by first addressing the overall electronically lowest transition and then stepwise moving toward lower wavelength.

For instance, Heckel and colleagues showed that extending the π‐system of *ortho*‐nitrobenzene (ONB)‐based PPGs allows to deprotect individually photocaged nucleobases in oligonucleotides in buffer chromoselectively. Specifically, irradiation with 440 nm cleaved the π‐extended nitrodibenzofuran (NDBF) first, and subsequent illumination with 365 nm light led to cleavage of the unsubstituted PPG [124].

**FIGURE 16:**
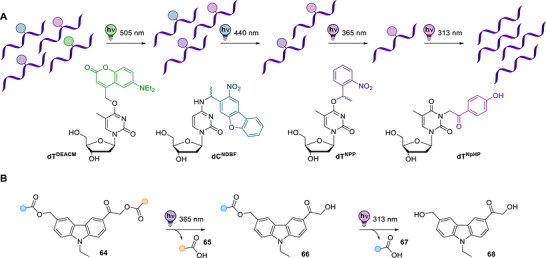
(A) Sequential release of four differently photocaged nucleobases. (B) Sequential dual release of two different cargos from the same chromophore.

Later, the same group could expand this concept and consecutively deprotect up to four individual PPGs caging different nucleobases (Figure 16A) [23]. Specifically, the authors synthesized dT^DEACM^ with a diethylaminocoumarin PPG, susceptible to irradiation with 505 nm light, dC^NDBF^ exhibiting NDBF, labile to 440 nm illumination, dT^NPP^ having 2‐(*ortho*‐nitrophenyl)propyl (NPP, responsive to 365 nm), and dT^NpHP^ with a *para*‐hydroxyphenacyl (pHP) PPG attached to the heterocyclic *N*‐atom, which can be removed with 313 nm light. The authors demonstrated that all photocaged nucleobases could be incorporated into DNA by solid‐phase synthesis. Melting experiments in the presence of an annealed complementary strand showed that the PPGs were effective in disturbing hybridization and lowering the melting temperature of the dsDNA. As the four photocages strongly overlap in their UV–vis absorption spectra toward the lower wavelengths, the compounds could be only sequentially uncaged, starting from the most bathochromically absorbing coumarin‐based one, followed by NDBF, NPP, and pHP. While the removal of the PPG by means of irradiation was successful, the authors noted the biological incompatibility of pHP, especially in the presence of DNA. This was due to the competing [2+2] photocycloaddition between two neighboring dT residues under the same conditions as required for pHP removal. While omitting neighboring dT in the sequence could circumvent this reactivity in artificial DNA nano‐systems, modulation in biological settings might be limited to the other three PPGs.

Using differently absorbing PPGs as caps for mRNA, Rentmeister and coworkers used light control of the latter in cellular studies [125]. Through transcription, a nucleic acid construct could be generated in vitro that was transfected into the target cells. Only upon illumination with UV light, the PPG‐cap was cleaved off from the mRNA, and translation was initiated. Further development of this strategy allowed co‐transfection of two constructs, each capped with a different PPG [126]. As their UV–vis absorption spectra differed, the coumarin‐based cap could be cleaved off with lower energy light than the ONB‐based one. In this way, either the translation of one (450 nm irradiation to only cleave the coumarin) or two (365 nm illumination to cleave both caps) proteins could be initiated in HeLa cells.

Sequential uncaging can also be used to activate different genetically encoded coumarin‐modified amino acids [127] or to precisely control the activity of a single biological target involving two BODIPY PPGs, one red‐light responding π‐extended derivatives and one unsubstituted core. By first releasing an agonist and then an antagonist targeting the same serotonin receptor 2C, the cellular response could be temporarily controlled, selecting both the starting and the end point [128].

To minimize the number of chromophores involved, chromophore engineering can enable the sequential release of two different cargo molecules from a single chromophore [129]. A carbazole chromophore, **64** (Figure 16B), was designed in such a way that one side exhibited a benzyl and the other one a phenacyl moiety. Since the two functional groups release their cargo following individual mechanisms [6], irradiation with 365 nm light triggered release from the phenacyl moiety, and subsequent irradiation with 290 nm light induced cleavage of the cargo from the benzyl site. The studies were performed in a MeCN/water mixture, indicating compatibility with aqueous media in general and thus, being a promising system to be used as a photocleavable linker or dual‐release PPG in a biological system, potentially optimizing the wavelength of irradiation to lower energy light. A similar strategy was applied to a hydroxynaphthalene, which showed conditional sequential release with the first uncaging event leading to a structural rearrangement allowing for a second cargo release at lower wavelengths [130].

The impact of chromoselective, wavelength‐dependent photolabile linkers was previously shown in the step‐wise softening of a hydrogel with three PPGs [131]. The three photolabile linkers selected were a *syn*‐tetramethyl bimane, an ONB, and a dimethyl aminobenzene. Through studying the action regime of the monomers, three distinct wavelengths, namely 420, 365, and 325 nm, were identified to address the bimane, the ONB, and the dimethyl aminobenzene units consecutively. The linkers were then embedded in a hydrogel‐forming polymer network, and the stepwise cleavage of the individual linkers, each with a different color of light, was reflected in a stepwise swelling of the hydrogel. This type of photoresponsive hydrogel still exhibited high levels of biocompatibility as pre‐osteoblast cells could be exposed to the polymer and the photodegradation process, and showed viability above 90% even 24 h after exposure.

Given the viability of the cells after several minutes of irradiation with blue and UV‐A light, the approach, as well as linker types, seems suitable also for other applications in a biological context. However, aspects of solubility and potential undesired reactivity of photo‐byproducts need to be considered (vide infra). While the control of three PPGs in a complex system offers a promising foundation, addressing these chromophores sequence‐independently, starting from low‐energy light and moving stepwise to increasingly higher energetic light, remains to be solved.

### 4.2 Two‐PPGs‐One‐Molecule Strategies

**FIGURE 17:**
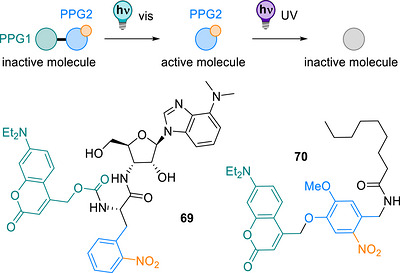
The Two‐PPGs‐One‐Molecule (TPOM) strategy was used to generate **69**, a translation inhibitor, and **70**, a ligand of the capsaicin channel. Low‐energy light first renders the molecules biologically active, while a second high‐energy light pulse deactivates them.

A more recent variation of chromoselective sequential uncaging was developed by combining two PPGs in one molecule, not with the aim of releasing two different cargos, but to activate the biological function of a small molecule reversibly. Wachtveitl, Schwalbe, and their teams modified the translation inhibitor puromycin so that it became susceptible to irradiation with UV‐C light by replacing the *para*‐OMe group on the phenyl moiety with an *ortho*‐NO_2_ one (orange and blue parts of **69**, Figure 17), while remaining biologically active [132]. Covalent attachment of a red‐shifted coumarin‐based PPG (green part in **69**) via a carbamate linker on the NH_2_ group rendered the construct inactive. In this way, the authors set the stage for a novel class of photopharmaceuticals, which they termed the Two‐PPGs‐One‐Molecule (TPOM) approach. In this molecule class, photons of lower energy (470 nm light to cleave the coumarin) will be used to activate the molecule. In contrast, a second, subsequent light pulse of higher‐energy light (365 nm to trigger the ONB) will be used to deactivate the molecule by photochemical cyclization into an inactive analogue. The authors could prove the practical use of their concept by using the TPOM puromycin that they developed to control the protein synthesis of GFP in a cell‐free coupled transcription−translation system.

Recently, Imse, Rojas et al. expanded the TPOM approach to capsaicinoids with the aim to reversibly activate the transient receptor potential cation channel subfamily V member 1 (TRPV1) [133]. While the two PPG‐units in molecule **70** (Figure 17) could be successfully cleaved consecutively, TRPV1 deactivation could not be implemented in cellulo, presumably due to the high sensitivity of the channel and the prolonged irradiation times with UV light.

### 4.3 Towards Sequence‐Independent Strategies

The key challenge in the development of fully *λ*‐orthogonal strategies is to be able to invert the order of chromophores irradiated, that is, being able to irradiate a chromophore in the high‐energy region of the electromagnetic spectrum with high chromatic selectivity η in the presence of other molecules absorbing lower energy light.

**FIGURE 18:**
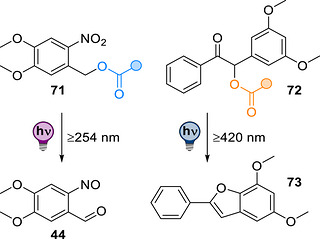
The seminal *λ*‐orthogonal pair of PPGs introduced by Bochet et al.

Already in 2000, Bochet et al. introduced a pair of PPGs that could be addressed in the presence of each other with acceptable selectivity (Figure 18) and shaped the term *λ*‐orthogonal reactivity with their seminal work [13, 14, 16]. Over the following two and a half decades, the concept has inspired applications from polymer chemistry to biology and has been expanded to diverse chromophores.

**FIGURE 19:**
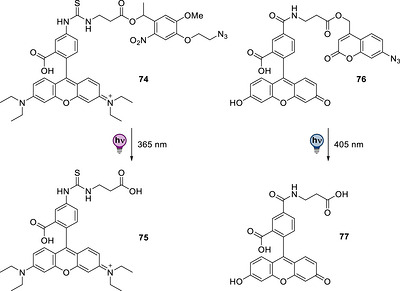
Rhodamine and fluorescein as model cargo conjugated to light‐sensitive PPG‐based linkers with azide anchors for incorporating the constructs into hydrogels. Later, the fluorophores were replaced by maleimide groups for protein conjugation.

For instance, Azagarsamy and Anseth combined an ONB and a coumarin photocage to release two different proteins from hydrogels selectively [134]. Using the release of fluorescein and rhodamine as model compounds (**74** and **76** in Figure 19), the authors identified 365 nm LED light as the preferred release of cargo from the ONB cage over the coumarin one, while 405 nm light showed the opposite trend. Azide groups attached to the core of the photocages enabled the incorporation of both chromophores into hydrogels. In contrast, the attachment of linkers with a maleimide end group at the photocleavable position of the cages facilitated conjugation to cysteine groups of two different proteins. While the hydrogel‐protein constructs stayed stable over days in the absence of light, irradiation with different colors of light released the respective proteins within a few minutes. The concept of the work is generalizable and has high biocompatibility; however, it has to be noted that due to the overlap of UV–vis absorption bands of the two chromophores, unintended background release of the wrong cargo could not be entirely avoided, and also, the proteins could not be released in their native form, due to the necessity of attaching a maleimide‐bearing linker.

Introducing a photolabile moiety as a linker into a DNA backbone, a team around Bolze, Specht, and Lo used the λ‐orthogonal cleavage of two different biohybrid constructs to generate a series of molecular logic gates [135]. As linkers, they employed a classical ONB, responsive to UV‐C light, and a π‐extended analogue, which could be cleaved with visible light (>420 nm via a long‐pass filter on a Xenon lamp). By solid‐phase DNA synthesis, the linkers were incorporated at various positions on the DNA strands, which were also functionalized with a FRET pair for visualization. In this manner, the selective cleavage with two different colors of light could be translated into a series of logical operations, depending on the positions of the fluorophores and the light applied.

**FIGURE 20:**
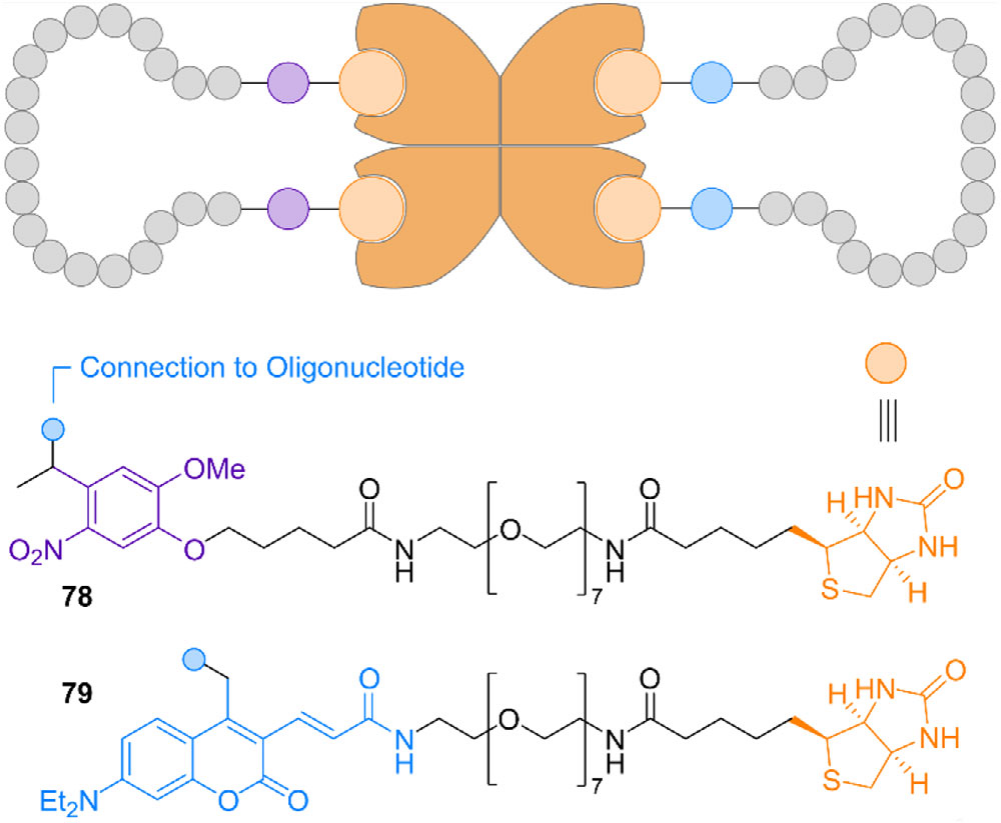
Oligonucleotides connected to PPG‐based linkers **78** or **79** could be handcuffed onto streptavidin (representation as clover‐shaped protein, top) via their biotin moiety (bottom). The use of different PPGs, that is, ONB (violet in **78**) and coumarin (blue in **79**), rendered the cleavage and liberation of the nucleic acids *λ*‐orthogonal.

Booth and coworkers used PPGs functionalized with biotin to “handcuff” antisense oligonucleotides (ASOs) as an alternative means to control nucleic acids (Figure 20) [136]. The PPGs were installed on both termini of the ASOs and connected to the same streptavidin protein. This supramolecular protection strategy showed significantly reduced biological activity of the ASOs. As PPGs, the authors used ONB **78** and coumarin **79**. Upon irradiation with UV (365 nm) or blue light (455 nm), respectively, the ASOs could be liberated from the supramolecular protecting group and regained their biological activity as RNase H against their target mRNAs under cell‐free conditions in aqueous media. The red‐shifted coumarin and the ONB turned out to be addressable in a *λ*‐orthogonal fashion. They were thus further developed into chromoselectively controllable DNA‐based tools and logic gates. By demonstrating the chromoselectivity in a mixture of both PPGs, each conjugated to a different ASO, irradiation with either violet or blue light led to degradation of only one of the two possible mRNAs with good selectivity [137].

Moreover, the authors could show that these tools can be used to both initiate and terminate protein expression in cell‐free systems [138]. In particular, they protected DNA‐templates with the same PPG‐biotin construct as introduced above, attaching the light‐responsive groups onto all dT residues in the T7 promotor region of a selected DNA. Thus, the promoter was not readable before activation through illumination and allowed for gene expression only after irradiation with light and photochemical deprotection. By attaching the ONB and the coumarin‐based linker onto two different DNAs, one leading to the expressing the α and the second to the expressing the ω subunit of the same protein, their chromoselective activation could be translated into a logic AND gate: only light‐activation with blue and violet light led to the expression of assembly of all protein subunits and thus, to catalytic activity. The same chemistry allowed for spatial control of gene expression in the 3D space in droplet‐based synthetic cells, demonstrating the appeal of using synergistically working multi‐photoactivated systems.

It was further possible to combine the two concepts, namely light‐mediated activation of gene expression and gene knockdown. Employing λ‐orthogonal PPGs, full photochemical control over the start and end of transcription and translation could be obtained in a cell‐free setting [137]. Since sequential release of nucleic acids from PPGs has been used to control translation (vide supra), orthogonal light control through strategies like there here‐shown handcuffed ASOs appear applicable once practical aspects, such as biocompatibility of the side products and delivery, are investigated.

**FIGURE 21:**
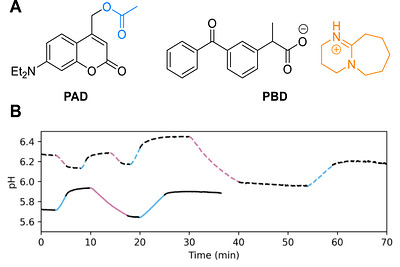
(A) Chemical structures of photoacid donor **PAD** and photobase donor **PBD**. (B) A solution of **PBD2** (0.40 mM) and **PAD3** (0.63 mM) in 50% methanol in water was irradiated with 450 nm (magenta line segment) and 310 nm (blue line segment) light.

Using a combination of two *λ*‐orthogonal PPGs to invert each other's actions could also be established, involving small molecules. Specifically, mixing a photoacid donor **PAD** and a photobase donor **PBD**, Chaudhuri et al. recently demonstrated that the pH of a given solution could be increased by addressing the **PBD** and decreased by triggering uncaging in the **PAD** in a sequence‐independent manner from various starting points (Figure 21) [139]. While this could be a promising approach to achieve control at the system level, a practical limitation might be the currently high‐energy light (310 nm) required to address the **PBD**.

High‐energy light is not only a concern for phototoxicity in biological samples, but it also faces inner‐filter effects by other chromophores present in the sample, like hemoglobin [140]. To overcome the usage of UV‐light irradiation for the (chromoselective) removal of PPGs, an increasing amount of attention was dedicated to the development of visible‐light responsive PPGs, based on, for instance, coumarin [141] or BODIPY scaffolds [26, 142, 143].

**FIGURE 22:**
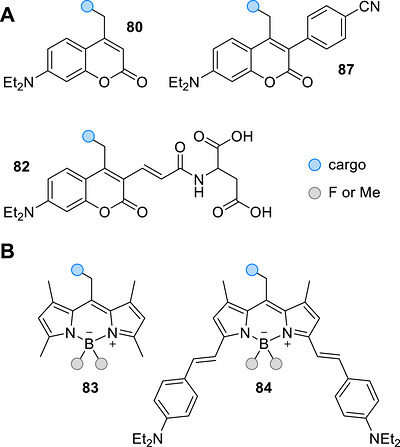
(A) Classical diethylaminocoumarin **80** and the π‐extended analogues **81** and **82**. (B) Classical (**83**) and π‐extended BODIPY (**84**); exchanging F with Me (grey spheres) at the boron additionally enhanced the QY.

Ellis‐Davies and their team, for example, extended 7‐aminocoumarins at the 3‐position to tune the UV–vis absorption spectrum of the molecules [141]. Indeed, introduction of nitro, cyano, or *para*‐cyano phenyl groups (Figure 22A), among others, led to a bathochromic shift in the absorbance and spectrally separated the lowest electronic transition bands. The PPGs were then used to cage various bioactive groups, such as phosphate, glutamate, and AMP, and the photochemically uncaging efficiencies in one‐ and two‐photon processes were compared in different biological settings.

Smith, Winter, and colleagues extended the π‐system of different BODIPY‐based PPGs to allow for visible and NIR light‐triggered photochemical bond cleavage (Figure 22B) [26]. Next, they studied different PPGs involving red‐shifted BODIPYs as well as coumarin‐based PPGs regarding their propensity to be released *λ*‐orthogonally or sequentially in the presence of each other. While sequential release worked in all combinations involving up to three different PPGs, *λ*‐orthogonal release could only be achieved in optimally engineered chromophores, capitalizing on the neat interplay of photouncaging QY and absorptivity. Exchanging fluoro with methyl substituents on the boron in BODIPYs enhances their QY and can be used as an additional handle to speed up or slow down photochemical uncaging.

### 4.4 The 3D Challenge

Light irradiation has been proven to result in high spatiotemporal resolution, with chromophores being only (de)activated where photons reach. While the previous section concluded with methods to enhance visible and NIR light absorptivity through chromophore design, addressing a 3D medium, such as a biological sample, with high precision poses a greater challenge than solving the issue of penetration depth. Spatiotemporal precision can only be fully unlocked if the area of irradiation can be limited to the one desired in the 3D space, and examples were already discussed for photoswitches (vide supra).

Heckel and colleagues addressed both aspects simultaneously by using low‐energy NIR light to achieve 3D resolution in a two‐photon (2P) approach [144]. By illuminating a sample from two different directions, the highest probability to simultaneously absorb two photons is at the crossing point, so that the uncaging could occur in this area.

**FIGURE 23:**
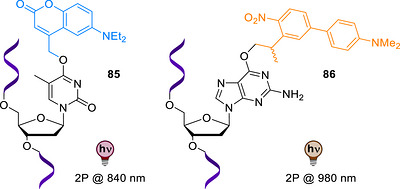
Using 2P photocages, the nucleobases in **85** and **86** could be protected and incorporated into synthetic DNA, so that the strands could only properly engage in hybridization after light‐induced uncaging.

To additionally realize *λ*‐orthogonality, the authors combined coumarin **85** (Figure 23) and extended ONB **86** as 2P‐responsive PPGs and used them to cage a dT or a dG residue, respectively, in synthetic DNA single strands [145]. Using a fluorescence‐based assay in a hydrogel, the authors could visualize the 2P uncaging events in the 3D space upon strand replacement of a quenching strand with the now liberated ssDNA, which resulted in fluorescence increase. The same experiment could be performed in cultured neurons and was then optimized by adjusting the 2P wavelengths in such a way that both photocages could be addressed individually. While irradiation at 840 nm with restricted power (1.3 mW) led to uncaging of **85**, employing 980 nm (8 mW) could selectively uncage **86**‐protected nucleobases in the model DNAs. Combining both wavelengths in a sequence or using 840 nm light at higher intensities (13 mW) resulted in photochemical cleavage of both protecting groups.

**FIGURE 24:**
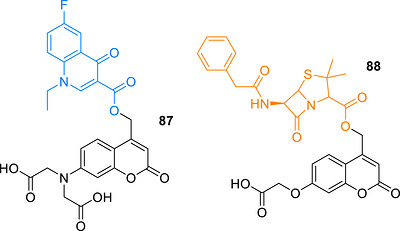
The photocaged antibiotics **87** and **88** could be liberated *λ*‐orthogonal in showed activity toward either Gram‐positive or Gram‐negative bacteria.

Feringa and coworkers showed that in a mixture of different antibiotics, each protected with a different PPG, uncaging could be achieved chromoselectively [146]. Specifically, fluoroquinolone was protected with a diethyl‐amino coumarin derivative (**87** in Figure 24), which absorbs more toward the red compared to **88**, which was used to act as PPG for benzylpenicillin. Thus, irradiation with visible light led to the liberation of fluoroquinolone and targeted *E. coli*, while 312 nm light could uncage benzylpenicillin and inhibit the growth of *S. aureus* on the same plate in a spatially resolved manner. Recently, Feringa, Szymanski, van Dijl, and coworkers expanded the same concept involving green‐light‐responsive PPGs, reducing potential concerns regarding photo‐toxicity [147]. Additionally, the authors used the two‐wavelength orthogonal activation to enhance the control of antimicrobial activity in the 3D space: only the simultaneous liberation of two different antibiotics, penicillin and tazobactam, inhibited bacterial growth in an agar plate.

### 4.5 PPGs in Complex Biological Environments and Challenges for Chromoselective Systems

Like photoswitches and photoclick agents, PPGs are based on organic chromophores and thus face similar challenges when it comes to their solubility in the aqueous buffer conditions required for cell and in vivo studies. Sufficient solubility can be either achieved by incorporating the PPG into a biomacromolecule, such as proteins or nucleic acids (vide supra), connecting it to a readily soluble small molecule, or by attaching solubilizing groups, which are especially important for the larger PPGs. For instance, derivatives from the BODIPY family have been shown to J‐aggregates, a property that is exploited for photodynamic therapy (PDT) [148, 149, 150], but might be hampering when designing PPGs.

**FIGURE 25:**
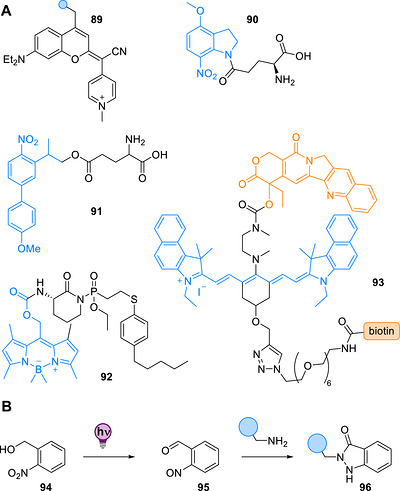
(A) Examples of biocompatible PPGs, for mitochondria‐targeting [141], neurobiological studies through the release of caged glutamate in 1P [152] and 2P processes [153], for uncaging of a covalent DPP8/9 inhibitor [154], and a dual‐channel fluorescent prodrug for in vivo studies [155]. (B) The nitroso‐benzaldehyde photoproduct of ONB can undergo a photoligation reaction with primary amines [6, 156, 157, 158].

Coumarin photocages offer various attachment points for solubilizing groups, which can also be combined with π‐extension approaches to red‐shift the UV–vis absorption spectrum. For instance, attaching a carboxylic acid group to the hydroxy or amino group attached to position 7 of the core facilitated enhanced solubility and led, for instance, to **87** and **88** being applied as antimicrobial agents (vide supra). Specifically, the compounds could be used to *λ*‐orthogonally prohibit growth of different bacterial cells in culture [146] and could even be moved forward for in vivo infection treatment, using red‐shifted analogous [147]. Also, modifications in position 2 [151] and 3 [141] of coumarins can lead to bathochromic shifts via π‐extension and enhanced solubility through the introduction of functional groups with the same modification. These modifications not only open possibilities for chromoselective control through spectral separation, but also directly led to applications in living cells, such as the targeted cargo‐realize in mitochondria (**89** for position 2 in Figure 25 and **82** in Figure 22A for position 3).

Next to solubility, the light‐induced excitation and bond cleavage reaction needs to proceed effectively toward cargo release, ideally without any secondary processes involved, and the obtained photoproducts need to be benign. All the above‐discussed PPGs, that is, ONBs, coumarins, and BODIPYs, have been shown to effectively release their cargos both in organic solvents and under aqueous buffer conditions. In particular, ONB photolysis depends on leaving group effects [159], the effectiveness of the excited‐state hydrogen transfer [160], the ground state reactivity of the quinoid intermediates, as well as solvent properties, including the pH of buffers [6, 161]. Interestingly, coumarins and BODIPY, which preferably cleave in an excited state solvolysis reaction for many derivatives, do this more effectively in polar protic environments, favoring cleavage in biological relevant media [162].

Since PPGs are well‐investigated for decades, they are likely the most developed light‐responsive chromophores for biological applications, besides agents for PDT, and several compounds have been established as powerful tools to study cell physiology [152] and for other applications in vivo [9, 11]. Besides caged calcium [163], the release of neurotransmitters, such as glutamate (**90** and **91**, Figure 25A), has been extensively studied in vitro [152], on neurons [153, 164], brain slices [165], and animal models, such as freely moving mice [166], showcasing the broad applicability of glutamate‐PPGs for biological studies, which are comprehensively reviewed elsewhere [152, 167].

Also, applications involving the release of other small, bioactive molecules have been demonstrated to work well in complex biological media [168]. For instance, compound **92** (Figure 25A) was used as a photo‐cleavable covalent DPP8/9 inhibitor and validated in cell studies [154]. On the other hand, **93** serves as a dual‐channel fluorescent prodrug that can release bioactive cargo triggered by NIR light. The multi‐modality of the compound allowed to precisely trace and control over drug release in vivo, shown in the mouse model [155].

Since individual PPGs could be operated early on in cells and in vivo, it is not surprising that chromoselective control could already be conducted in complex biologically relevant settings (vide supra). Chromoselective uncaging of nucleic acids was realized both in cell‐free systems as well as synthetic cells [124, 135, 136, 137] and for translation control in living cells [125, 126]. Multi‐wavelength control in hydrogels allowed to uncage anchored DNA with 2P processes and 3D resolution in solution and on neurons [144, 145] or to modulate the growing environment in the presence of cells, to only highlight a few examples from the previous section [159].

Naturally, introducing precision photochemistry to in vivo systems appears to be the next logical step, so that additional considerations regarding biocompatibility need to be taken. In vitro and cell studies showed that, for instance, green and red‐light responding BODIPY PPGs can induce ROS and thus lead to phototoxicity [169], a finding that is not too surprising since some BODIPYs are considered suitable agents for PDT [148, 149, 150]. Also, thiocarbonylated coumarins showed suitability for PDT [170]. Depending on the biological effect of the released cargo and the used PPG, the potential ROS generation might be negligible or aid the intended biological outcome. However, suitable controls need to be performed to correctly assign a biological response to cargo release.

While coumarin and BODPIY PPGs typically result in the corresponding alcohols or ether as photoproducts and are beyond potential excited state chemistry (ROS generation, fluorescence) considered as begin, ONBs yield nitroso carbonyls as byproducts after uncaging. Especially, electron‐deficient nitroso aldehydes react with primary amines, such as lysine, in a ligation reaction forming indazoles (cf. **96** in Figure 25B) [6, 156, 157, 158]. This reactivity can be largely avoided by using the in PPGs more frequently applied methoxy‐substituted derivatives or ONB PPGs, which yield a ketone, however, it should be considered when designing chromoselective sets of compounds for cell studies or in vivo settings.

## 5 Future Perspective and Challenges

Over the past decades, an increasing number of combinations of chromophores employing photoswitching, photoclick, or photouncaging chemistry have been engineered to operate chromoselectively. The systematic study of action regimes, together with defining the parameters *Φ*(*λ*), *ε*(*λ*), *c*(*t*), and *t* contributing to chromoselectivity *η*(*λ*,*t*), aids in enhancing the predictability in choosing sets of sequence‐specific or *λ*‐orthogonal light‐sensitive molecules.

Also, many fundamental studies on chromophore design extended the sets of light‐sensitive molecules available for different purposes and revealed crucial structure–property relationships when assembling (pairs of) molecules. For instance, in photochromic dyads, the *meta*‐connectivity rule was established to decouple the individual chromophores in their responsiveness to light. This empirical rule may be extended to generally disconnecting π‐conjugated systems, as demonstrated for *para*‐conjugated photoswitches, which were forced out of the same plane by sterically demanding *ortho*‐substituents. In bifunctional PPGs, the underlying uncaging mechanism also appeared to contribute to selectivity.

In all chromophores discussed, π‐extension approaches and substituent effects enabled the enhancement of band separation in the energetically lower‐lying electronic transitions, allowing for the design of sequence‐specific, chromoselective light‐sensitive molecules.

In multi‐stimuli responsive and exclusively *λ*‐orthogonal systems, additional factors, such as the (wavelength‐dependent) QY, as well as competing mechanisms, such as unintended thermal isomerization in the background of meta‐stable photoswitches, become essential. Indeed, the photoreaction of two light‐sensitive molecules absorbing at the same wavelength could be resolved (kinetically), resulting in sequential release if they differ significantly in their QY. Vice versa, *λ*‐orthogonality can be achieved if a short‐wavelength light‐absorbing chromophore with high QY is combined with a long‐wavelength light, low QY molecule. Importantly, precision photochemistry has shown that variations in concentration over time or in non‐equimolar settings also influence *η*(*λ*,*t*). In complex biological media, the competition for light with naturally occurring chromophores will contribute to selectivity profiles at time‐dependent concentration changes. Thus, determining or estimating the concentration of the chromophore at the target area, its change over time, and thus identifying a suitable end point of irradiation that provides a pronounced biological effect while realising high *η*, will be of key importance.

While the increasing predictability in the tunability of light‐sensitive molecules has led to immense progress in the development of chromoselective processes, not all light‐sensitive molecules have advanced comparably across diverse disciplines. In the context of this article, not all well‐functioning sets of chromoselective compounds can be directly applied to control biological systems. Several challenges remain to be tackled, and a larger number of in‐depth studies need to be conducted to unlock the full potential that λ‐orthogonal control offers for biology, but also for other applications.

On the one hand, π‐extension and substituent design have shown promising results to generate sequence‐dependent systems. However, expanding the organic core structure typically has a negative impact on aqueous solubility and can directly affect biological applications. Thus, a general approach to realise synthetically accessible, visible light‐responsive light‐sensitive molecules, while balancing aqueous solubility, needs to be developed.

Additionally, the action regime, that is, the interplay of absorbance and QY at different wavelengths, is known only for a limited number of light‐sensitive molecules. However, these parameters are key to predicting chromoselectivity and combining molecules into chromoselective ensembles [12]. Thus, a larger number of different light‐sensitive molecules needs to be systematically studied at different wavelengths and in different (biologically relevant) solvents. This may also enhance the number and types of different light‐sensitive molecules that can be combined within the same system and still be addressed selectively.

Lastly, a biological sample (as many other samples in an applied scenario) is a complex, three‐dimensional medium. Thus, truly unlocking the potential of light to induce discrete photochemical transformations spatiotemporally resolved requires finding more solutions to define a target area in 3D. Simultaneously, the presence of other (marco)molecules, like nucleic acids or proteins to which chromophores could potentially “stick” to, needs to be tolerated; likewise, cellular antioxidants, like glutathione, isomer‐dependent membrane partitioning, changes in the thermal half‐life or efficiency of excited state mechanisms under physiological conditions as well as phototoxicity, toxicity of photoproducts, or simply delivery of the chromophore to the target cell or organelle in cellulo and in vivo need to be considered.

Extrapolating from the potential already unlocked by my mono‐chromatically operating systems and the creativity that was already employed to realise chromoselective control in vitro and in biological systems, highlighted in a selection of illustrative examples here, the developments that we are going to see in the future will likely be of a transformative nature. They will pave the way for a selective, well‐controlled, and diverse toolbox to study, build, and manipulate biological systems in vitro and in vivo.

## Conflicts of Interest

The author declares no conflicts of interest.

## Data Availability

Data sharing is not applicable to this article as no new data were created or analyzed in this study.
